# RETRACTED: Chen et al. Inhibition of *AKT2* Enhances Sensitivity to Gemcitabine via Regulating *PUMA* and *NF-κB* Signaling Pathway in Human Pancreatic Ductal Adenocarcinoma. *Int. J. Mol. Sci.* 2012, *13*, 1186–1208

**DOI:** 10.3390/ijms27146511

**Published:** 2026-07-22

**Authors:** Dong Chen, Min Niu, Xuelong Jiao, Kejun Zhang, Jun Liang, Dianliang Zhang

**Affiliations:** 1 Department of General Surgery, The Affiliated Hospital of Medical College, Qingdao University, Qingdao 266003, Chinajiaoxuelong@163.com (X.J.);; 2 Department of Pharmacy, The Affiliated Hospital of Medical College, Qingdao University, Qingdao 266003, China; 3 Department of Oncology, The Affiliated Hospital of Qingdao Medical College, Qingdao University, Qingdao 266003, China

The Journal retracts the article “Inhibition of *AKT2* Enhances Sensitivity to Gemcitabine via Regulating *PUMA* and *NF-κB* Signaling Pathway in Human Pancreatic Ductal Adenocarcinoma” [1] cited above.

Following publication, concerns were brought to the attention of the Editorial Office regarding the reliability of the figures presented in this article [1].

In accordance with the journal’s standard procedures, the Editorial Office and Editorial Board conducted an investigation, which identified indications of inappropriate image editing and duplication in Figures 1 and 5. Despite multiple attempts to contact the authors and their affiliated institution, no response was received. In the absence of clarification from the authors and access to the underlying data required to verify the reported results, the Editorial Board has lost confidence in the integrity and reliability of the findings and therefore decided to retract this article in accordance with MDPI's retraction policy.

This retraction was approved by the Editor-in-Chief of the journal *International Journal of Molecular Sciences*.

The authors have been informed of this retraction but did not provide a comment on this decision.
